# Mind the Clinic-Community Gap: Re-evaluation of Test Performance and False Positive Results in Community-Wide Tuberculosis Screening

**DOI:** 10.1093/infdis/jiaf268

**Published:** 2025-05-23

**Authors:** Lara D Veeken, Alvaro Schwalb, Katherine C Horton, Raspati C Koesoemadinata, Bachti Alisjahbana, Reinout van Crevel, Rein M G J Houben

**Affiliations:** Department of Internal Medicine and Radboud Community for Infectious Diseases, Radboud University Medical Center, Nijmegen, The Netherlands; TB Modelling Group, TB Centre, London School of Hygiene and Tropical Medicine, London, United Kingdom; Department of Infectious Disease Epidemiology, London School of Hygiene and Tropical Medicine, London, United Kingdom; TB Modelling Group, TB Centre, London School of Hygiene and Tropical Medicine, London, United Kingdom; Department of Infectious Disease Epidemiology, London School of Hygiene and Tropical Medicine, London, United Kingdom; Instituto de Medicina Tropical Alexander von Humboldt, Universidad Peruana Cayetano Heredia, Lima, Peru; TB Modelling Group, TB Centre, London School of Hygiene and Tropical Medicine, London, United Kingdom; Department of Infectious Disease Epidemiology, London School of Hygiene and Tropical Medicine, London, United Kingdom; Research Center for Care and Control of Infectious Disease, Universitas Padjadjaran, Bandung, Indonesia; Research Center for Care and Control of Infectious Disease, Universitas Padjadjaran, Bandung, Indonesia; Department of Internal Medicine, Dr Hasan Sadikin General Hospital, Bandung, Indonesia; Department of Internal Medicine and Radboud Community for Infectious Diseases, Radboud University Medical Center, Nijmegen, The Netherlands; Centre for Tropical Medicine and Global Health, Nuffield Department of Medicine, University of Oxford, Oxford, United Kingdom; TB Modelling Group, TB Centre, London School of Hygiene and Tropical Medicine, London, United Kingdom; Department of Infectious Disease Epidemiology, London School of Hygiene and Tropical Medicine, London, United Kingdom

**Keywords:** tuberculosis, community screening, false positive diagnosis, specificity, Xpert MTB/RIF (Ultra)

## Abstract

Community-wide screening for bacteriologically confirmed pulmonary tuberculosis may reduce the tuberculosis burden, although concerns of overtreatment remain because of false positive diagnoses due to subpar specificity of current bacteriological tests for screening. Our review and data analysis show that clinic-based test specificity estimates of Xpert against culture underestimate performance in communities, for both Xpert MTB/RIF(99.8% for community vs 98.4% for clinic) and Xpert Ultra (99.4% vs 95.6%, respectively), reducing the presumed false positivity of sputum Xpert, using culture as the reference, by 86.8% and 85.4%, respectively, compared with clinic-based specificity estimates. These findings support large-scale evaluation of community-wide screening for tuberculosis.

## Teaser

This study shows that we likely overestimate the number of false positive tuberculosis diagnoses if clinic-based estimates of Xpert performance against culture are applied to community-wide tuberculosis screening, supporting the use of Xpert in evaluating community-wide screening for tuberculosis.

Tuberculosis is the world's deadliest infectious disease, with an estimated 1.25 million attributable deaths in 2023. The tuberculosis incidence remains high, with 10.8 million individuals acquiring the disease in 2023 [1]. A key challenge in ending tuberculosis is that about half of individuals with bacteriologically confirmed tuberculosis in national prevalence surveys do not report tuberculosis-suggestive symptoms (so-called asymptomatic tuberculosis) [2]. These individuals, who are likely infectious, do not seek care, and the current passive case finding system has therefore failed to stop *Mycobacterium tuberculosis* transmission [3], while symptom-agnostic community-wide screening is able to do so [4].

Mass screening in communities brings concerns on potential overtreatment as a result of false positive tuberculosis diagnoses due to subpar specificity of diagnostic tests for screening [5–7]. Besides unnecessary treatment, false positive diagnoses also affect households of individuals with tuberculosis, healthcare systems, and surveillance data [7]. Perceived risks of overtreatment contribute to the current World Health Organization (WHO) Global TB Programme screening guidelines recommending community-wide screening only in populations with a prevalence of ≥500/100 000 [5].

Conversely, the magnitude of false positive diagnoses might be overestimated when relying on the recorded performance of bacteriological tests as evaluated in clinic attendees. A negative association between specificity and prevalence exists, which likely reflects so-called “spectrum bias” [8], where the a priori risk of bacteriologically confirmed tuberculosis is lower because of the unenriched populations screened in community-wide studies compared with enriched populations, such as individuals presenting to healthcare facilities with symptoms, or symptom and/or chest radiographic screen–positive individuals in prevalence surveys. This should be factored into decisions on the design and implementation of community-wide tuberculosis screening. Here we compare the specificity of GeneXpert MTB/RIF (Xpert MTB/RIF) and GeneXpertMTB/RIF Ultra (Xpert Ultra) as an initial screening test in unenriched community-wide studies with the performance among enriched populations in prevalence studies and clinic-based settings.

## METHODS

We reviewed and extracted test performance data of screening studies in the general population, excluding high-risk populations, using Xpert assays (see Supplementary File 1 for our review strategy), widely implemented WHO-recommended rapid diagnostic tests. We searched the literature available in PubMed for studies that used sputum-based Xpert as an initial tool for bacteriologically confirmed tuberculosis screening in an unenriched community but also performed sputum culture testing. We also included prevalence surveys that performed both sputum-based Xpert and culture in screen-positive individuals (with symptoms and/or chest radiographic abnormalities). To compare Xpert performance in these studies with clinic-based performance, we used the diagnostic accuracy of Xpert compared with culture among individuals with presumed pulmonary tuberculosis in primary care facilities and local hospitals reported in a review by Zifodya et al [9].

The sensitivity (if possible) and specificity of sputum Xpert were determined, with sputum culture used as the reference. Standard methods for sensitivity and specificity with Wilson confidence intervals (CIs) were used for prevalence survey data, since sputum from all screen-positive individuals was tested with both culture and Xpert. Pooled estimates were calculated with Wilson CIs. In community studies, sputum culture testing was done only for individuals with a positive Xpert result, resulting in the absence of true negative and false negative numbers. For this reason, standard methods could not be applied, and the specificity of Xpert in community studies was calculated as follows:


$$\begin{array}{rl} & \mathrm{Specificity}=1-\;\mathrm{probability}\;\mathrm{of}\;\mathrm{returning}\;a\;\mathrm{positive}\\ & \;\;\;\mathrm{Xpert}\;\mathrm{test}\;\mathrm{result}\;\mathrm{if}\;\mathrm{culture}\;\mathrm{negative}, \end{array}$$

$$\mathrm{Specificity}=1-\frac{\mathrm{No}.\;\mathrm{false}\;\mathrm{postive}}{\mathrm{Estimated}\;\mathrm{No}.\;\mathrm{of}\;\mathrm{individuals}\;\mathrm{with}\;\mathrm{negative}\;\mathrm{culture}},\mathrm{and}$$

$$\begin{array}{rl} \mathrm{Specificity}=1 & -\frac{\mathrm{No}.\;\mathrm{false}\;\mathrm{postive}}{\mathrm{No}.\;\mathrm{individuals}\;\mathrm{consented}\;\text{–}\;}\\ & \left(\mathrm{No}.\;\mathrm{true}\;\mathrm{positive}/\mathrm{sensitivity}\;[\text{\%}]\;\times 100\right). \end{array}$$

(See Supplementary File 1 for rationale and derivation, and R packages used.)

We calculated the specificity of the Xpert Ultra in 2 ways: (1) classifying trace results as Xpert Ultra positive, as primary analysis, and (2) classifying trace results as Xpert Ultra negative. A false positive diagnosis was defined as a positive Xpert result with a paired negative culture result.

We determined the number of false positive diagnoses per 1 true positive diagnosis by Xpert as reported by the studies, and we also estimated this ratio for each of the studies, assuming a sputum culture-positive tuberculosis prevalence of 500/100 000 (0.5%) [5]. Moreover, we determined the proportion of false positives among all Xpert-positive results (Supplementary File 1). Finally, we performed a sample size calculation for a community-wide population study establishing test performance in terms of specificity and sensitivity in a community with a sputum culture-positive tuberculosis prevalence of 0.5%.

## RESULTS

**Figure 1.**
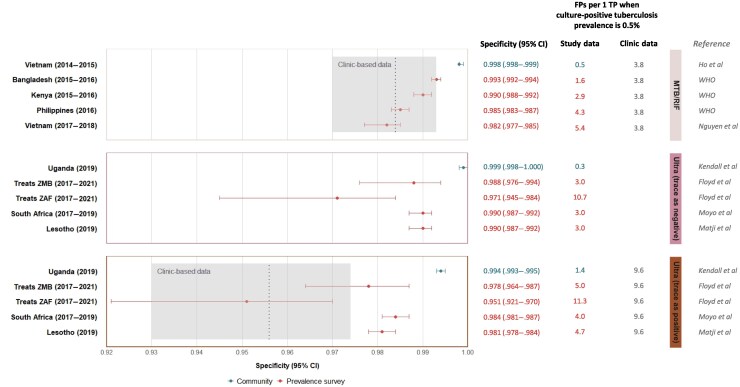
Xpert test performance with culture as the reference standard. Specificities are displayed for Xpert MTB/RIF (*top panel*), Xpert Ultra with trace results classified as negative (*middle panel*), and Xpert Ultra with trace results as positive (*bottom panel*), among screen-positive individuals in prevalence surveys and individuals in the community, compared with clinic settings. The number of false positive diagnoses (FPs) per 1 true positive diagnosis (TP) are also displayed, with the estimated specificity and sensitivity applied to a culture-positive tuberculosis prevalence of 500/100 000. Dashed lines represent point estimates; gray areas, 95% credible intervals (CrIs) from clinic-based estimates by Zifodya et al [9]: sensitivity (95% CrI) and specificity (95% CrI), 0.847 (.786–.899) and 0.984 (.970–.993), respectively, for Xpert MTB/RIF and 0.909 (.862–.947) and 0.956 (.930–.974) for Xpert Ultra. Abbreviations: CI, confidence interval; WHO, World Health Organization; Treats ZAF, treats study in South Africa; Treats ZMB, treats study in Zambia. See Supplementary Table 1 for references.

We included published data from community-wide screening studies in Vietnam [10] and Uganda [11], where sputum Xpert MTB/RIF (Vietnam) or Xpert Ultra (Uganda) were used to screen all adults. We also included data from 8 prevalence surveys that used sputum Xpert MTB/RIF (50%) or Xpert Ultra (50%) for confirmatory testing of adults who screened positive based on symptoms and/or chest radiography (Supplementary Table 1). In a Vietnamese community with an estimated prevalence of culture-positive tuberculosis of 0.3%, the Xpert MTB/RIF specificity was 99.8% (95% CI, 99.8%–99.9%) [10 ], substantially higher than the clinic-based estimate of 98.4% (95% credible interval [CrI], 97.0%–99.3%) [9]. Similarly, in a Ugandan community with an estimated culture-positive prevalence of 0.4%, the specificity of Xpert Ultra (with trace results classified as positive) was 99.4% (95% CI, 99.3%–99.5%) [11], far exceeding the clinic-based estimate of 95.6% (95% CrI, 93.0%–97.4%) [9]. When trace results were classified as negative, the Xpert Ultra specificity further increased to 99.9% (95% CI, 99.8%–100%) (Figure 1 and Supplementary Tables 2–4).

In prevalence surveys, the Xpert test specificity among individuals positive at screening often overlapped with the 95% CrI of clinic-based estimates. The pooled specificity was 98.8% (95% CI, 98.2%–99.2%) for Xpert MTB/RIF, and 97.7% (95% CI, 96.3%–98.6%) for Xpert Ultra (with trace results classified as positive) (Figure 1). The pooled sensitivity of Xpert MTB/RIF was 73.2% (95% CI, 63.3%–81.3%) compared with culture and 84.7% (95% CrI, 78.6%–89.9%) in clinics [9] (Supplementary Table 5). For Xpert Ultra, the pooled sensitivity was 82.5% (95% CI, 75.9%–87.6%) when trace results were classified as positive compared with 90.9% (95% CrI, 86.2%–94.7%) in clinics [9] and 65.3% (95% CI, 57.8%–72.1%) when trace results were classified as negative.

In a community with a prevalence of culture-positive tuberculosis of 500/100 000, we estimated false positive to true positive ratios of 0.5:1 for Xpert MTB/RIF and 1.4:1 for Xpert Ultra (with trace results classified as positive) (Figure 1). These ratios are 86.8% and 85.4% lower, respectively, than the false positive to true positive ratios of 3.8:1 for Xpert MTB/RIF and 9.6:1 for Xpert Ultra (with trace results classified as positive), estimated among individuals with presumptive tuberculosis reporting to a clinic. In prevalence surveys, these ratios among screening-positive individuals were not consistently lower than estimates derived from clinic-data and showed less deviation from their respective clinic-based estimation (Figure 1 and Supplementary Table 6). In a community with a sputum culture–positive tuberculosis prevalence of 0.5%, the required sample size would be 5757 to confirm an Xpert test specificity of 99.4% and 1927 for a specificity of 99.8%, with a 95% CI width of ±0.2 (Supplementary Tables 7–8).

## DISCUSSION

This analysis shows how we overestimate the expected number of false positive diagnoses if clinic-based estimates of Xpert performance against culture are applied to scenarios of community-wide tuberculosis screening. In a community with a culture-positive tuberculosis prevalence of 500/100 000, we estimated that clinic-based specificity values would lead to a 7-fold overestimation of the number of false positive Xpert Ultra diagnoses relative to each true positive diagnosis. Relying on clinic-based Xpert specificity in decision making and mathematical modeling could significantly underestimate the positive impact of Xpert community-wide screening in reducing the tuberculosis burden.

The high specificity of Xpert observed in community-wide screening studies limits the negative impact of false positives and strengthens the rationale for implementing a community-wide screening. Previously stated concerns about an expected high number of false positive Xpert diagnoses in prevalence surveys align with our findings [6, 7]. However, we are at risk of strongly overestimating the negative effects of community-wide screening with Xpert as initial screening if we apply clinic-based specificity. Notably, the increased specificity of Xpert in a community setting is characterized by lower overall sensitivity. This decline is expected, since individuals in a community are more likely to present less severe states of disease than those in clinic settings, which is referred to as “spectrum bias” in diagnostic accuracy studies [8]. Nevertheless, the overall reduced sensitivity minimally affects the beneficial epidemiological impact of screening [7], and sensitivity remains high among those with the most severe and infectious forms of tuberculosis [8].

To support implementation of community-wide Xpert screening in countries with a high tuberculosis burden, we must better understand why some individuals have positive sputum Xpert results but negative sputum cultures and whether this has implications for treatment. First, bacteriologically confirmed tuberculosis could have been correctly captured by Xpert but missed by culture, since the performance of culture is affected by sputum quality, sputum handling, and the number of sputum specimen cultured [12], likely underestimating the number of individuals with viable *M. tuberculosis* in their sputum [13]. Second, in high-burden settings, Xpert might be more sensitive to the high prevalence of biologically irrelevant *M. tuberculosis* colonization or nonreplicating *M. tuberculosis* in the respiratory tract than sputum culture. In South Africa, approximately 90% of individuals with presumed tuberculosis produced *M. tuberculosis*–containing bioaerosols, compared with approximately 80% of randomly recruited individuals in the community [14]. However, since tuberculosis exists on a spectrum, a positive Xpert test result with a negative culture likely indicates a high risk of progression to culture-positive disease, suggesting that such individuals could still benefit from treatment.

Third, false positive results can be caused by sample processing methods, including increased cross-contamination risk in clinics relative to lower prevalence community settings. Fourth, individuals who have recovered, either naturally or through treatment, may return a positive Xpert result, due to residual mycobacterial DNA [15]. In community settings, the proportion of individuals with a history of tuberculosis might be lower than in clinics, therefore reducing the probability of false positives. This phenomenon is more likely for “trace positive” Xpert Ultra results and could explain why the specificity of Xpert MTB/RIF is more similar to that of Xpert Ultra if trace results are classified as negative [13]. To reduce overtreatment even further, we must consider the potential importance of clinical evaluation after a positive Xpert screening result (eg, tuberculosis-suggestive symptoms, chest radiographic findings, an epidemiological indication, or a response to broad-spectrum antibiotics) and how this varies by a priori risk of bacteriologically confirmed tuberculosis and factors leading to a positive Xpert test result despite a negative culture.

The main limitation of this study was the unknown number of true and false negatives in community studies due to the lack of sputum culture testing for individuals with a negative sputum Xpert result. Nevertheless, our analysis indicates that the use of Xpert as an initial screening test might be a better community screening strategy than previously considered. This highlights the significant value of information in extending validation studies beyond the clinic and into the community, confirming the high specificity of (new) bacteriological screening tests, which is notably smaller than the size needed to establish sensitivity. Notably, future studies should carefully consider the criteria for “good-quality” sputum samples and how these affect diagnostic yield. In addition, prevalence survey data were limited by variations in methods, incomplete sensitivity of chest radiographic screening, and missing or contaminated cultures. Most importantly however, surveys uniformly show that focus on passive case finding alone will leave undiagnosed and untreated a major proportion of individuals with bacteriologically confirmable tuberculosis [2].

Our work highlights the clinic-community gap, where Xpert demonstrates substantially higher specificity as an initial screening tool in community settings compared with its use in clinics. As community-wide symptom-agnostic screening is the only tool currently supported by evidence to effectively reduce the prevalence of bacteriologically confirmed tuberculosis [4], the evaluation of the potential benefits and harms of screening approaches should use high-quality data on test performance. We should be concerned about overtreatment, but we must be able to rely on appropriate data on test performance to determine the magnitude of overtreatment, assess cost-effectiveness, and inform epidemiological modeling studies. Therefore, studies should also establish the specificity of potential screening tests in the community, rather than in clinic populations.

## Supplementary Material

jiaf268_Supplementary_Data

## Supplementary Data

Supplementary data are available at *The Journal of Infectious Diseases* online (http://jid.oxfordjournals.org/). Supplementary data consist of data provided by the author that are published to benefit the reader. The posted materials are not copyedited. The contents of all supplementary data are the sole responsibility of the authors. Questions or messages regarding errors should be addressed to the author.
