# Supplementary material for: Mind the Clinic-Community Gap: Re-evaluation of Test Performance and False Positive Results in Community-Wide Tuberculosis Screening
Source: J Infect Dis. 2025 May 23;232(2):e242–6. doi: 10.1093/infdis/jiaf268 (PMC12349932; doi:10.1093/infdis/jiaf268)
Supplement: jiaf268_Supplementary_Data [file jiaf268_supplementary_data.zip › Veeken2025_AppendixForReviewers.pdf]

Appendix for reviewers for *Mind the clinic-community gap: re-evaluation of test performance and false positive results in community-wide tuberculosis screening*

Table of contents

Introduction 1
Table 1. Data community-wide tuberculosis screening in Viet Nam & Uganda 1
Method calculation specificity Viet Nam by Ho *et al*. (2016) [1] 2
Exercise to derive the specificity formula by Ho *et al*. [1] 3
Method calculation specificity Uganda by Kendall *et al*. (2021) [2] 4
Exercise to derive the specificity formula by Kendall *et al*. [2] 5
Decision on the final method used in this study 5
Table 2. Comparing estimated specificity with the three different methods 7
References 8

Introduction

Ho *et al*. [1], and Kendall *et al*. [2] have previously estimated the specificity of their community-wide screening studies. The aim of this appendix is to compare the methods for estimating specificity, in absence of false negative and true negative numbers. First, we show a table with paired Xpert and culture results of the two community-wide screening studies. For Viet Nam, we used the data from Ho *et al*. (2016) [1], in which they estimated the specificity based on results of the first year of the ACT3 trial. Additionally, we note that there are some discrepancies between the data of the first intervention year used by Ho *et al*. (2016) [1] and finally presented at the end of the ACT3 trial by Marks *et al*. (2019) [3]. Second, we show and derived the method by Ho *et al*. [1] to estimate the specificity of Xpert MTB/RIF. Third, we show and derived the method by Kendall *et al*. (2021) [2] to estimate the specificity of Xpert Ultra. Fourth, we compared the specificity estimations using all three methods, and elaborated on our method choice.

Table 1. Data community-wide tuberculosis screening in Viet Nam & Uganda

	**Vietnam – ACT3**		**Uganda – Kendall *et al.* [2]**
	**Ho *et al.* [1] (Intervention year 1)**	**Marks *et al.* [3] (intervention year 1)**	
Xpert positive and culture positive (true positive)	94	95	35
Xpert positive and culture negative (false positive)	60	63	59
Xpert positive and culture not done	15	11	19
Xpert positive (total)	169	169	113
Consented	43435	43425	12301
Able to produce sputum for Xpert testing	23202	23282	-
Valid Xpert results	22673	-	12032

Method calculation specificity Viet Nam by Ho *et al*. (2016) [1]

<u>Described specificity calculation</u>

Specificity = number of true negatives / (1–prevalence)

(1–prevalence) – number of false positives

Number of true positives × (1–positive predictive value)/positive predictive value

prevalence x sensitivity

<u>This was calculated with two options for 'prevalence'</u>

Prevalence = all positive Xpert MTB/RIF results / denominator

With all positive Xpert MTB/RIF results

Numerator 'prevalence' = all positive Xpert MTB/RIF results

= TP + FP + positive Xpert with missing culture

= $xpert_{pos}cult_{pos}$ + $xpert_{pos}cult_{neg}$ + $xpert_{pos}cult_{missing}$ = 94 + 60 + 15 = 169

With two options for the denominator

1) Consenting participants (43435)
   - ➔ Resulting in a prevalence of positive Xpert MTB/RIF results among consenting participants of: 169/43435*100 = 0.39%
   - ➔ This assumes that all participants with a negative Xpert MTB/RIF result would have had a negative sputum culture result if tested, and thus are true negative.
2) Participants with valid sputum Xpert MTB/RIF results (22673)
   - ➔ Resulting in a prevalence of positive Xpert MTB/RIF results among participants with a valid sputum Xpert MTB/RIF results of: 169/22673*100 = 0.75%

<u>This was calculated with two options for the positive predive value</u>

1) PPV = 61%
   - ➔ Xpert MTB/RIF compared against culture = ($xpert_{pos}cult_{pos}$ / Xpert positive with culture done)
     = 94/154 (61.0%, 95% CI 52.8–68.7)
2) PPV = 83.9%
   - ➔ Xpert MTB/RIF compared against culture and/or CXR = 125/149 (83.9%, 95% CI 76.8–89.2)

<u>This was calculated with two options for sensitivity</u>

1) 89% median pooled sensitivity of Xpert MTB/RIF from a 2014 meta-analysis [4]
2) 63% is the lower end of the 95th credible interval

➔ Note: a more recent systematic review by Zifodya *et al*. (2021) [5] estimated Xpert MTB/RIF sensitivity of 84.7% (95% CI: 78.6 to 89.9), 63% does not fall within the 95% confidence interval.

Exercise to derive the specificity formula by Ho *et al*. [1]

1) Specificity = number of true negatives / (1–prevalence)

Specificity = TN / (1 – (TP + FP + positive Xpert with missing culture)

Specificity = TN / (1 – ($xpert_{pos}cult_{pos}$ + $xpert_{pos}cult_{neg}$ + $xpert_{pos}cult_{missing}$))

Compared to <u>standard specificity</u> formula:

Specificity = TN / (TN + FP)

Specificity = TN / (1 – (FN + TP)) *with the assumption that 1 = TN + FN + TP + FP, so TN + FP = 1 – (FN + TP)*

Specificity = $xpert_{neg}cult_{neg}$ / (1 – ($xpert_{neg}cult_{pos}$ + $xpert_{pos}cult_{pos}$))

The denominator for specificity should consist of all individuals tested with a negative reference sputum culture, which could be determined by subtracting the number of all culture positive (FN + TP) individuals from the number of Xpert tested individuals (TN + FN + TP + FP). However, the number FN ($xpert_{neg}cult_{pos}$) is unknown. To deal with this missing information, it seems like Ho *et al*. [1] subtracted the proportion FP + $xpert_{pos}cult_{missing}$ instead of TN. However, the sum of 'FP + $xpert_{pos}cult_{missing}$= 75' is likely to be <u>lower</u> than the number of FN (depending on the negative predictive value [NPV]†). This might have resulted in an overestimated number of individuals with a negative reference sputum culture (TN + FP) if all individuals would have been tested for culture.

† Calculation why it is likely that the sum of 'FP + $xpert_{pos}cult_{missing}$= 75' is lower than number of FN:

Calculation

false negatives = $Xpert_{negative}$* (1-NPV)

false negatives = (denominator – Xpert positive)*(1-NPV)

**Scenario§**	FN with consenting participants as denominator	FN with participants with valid Xpert MTB/RIF result as denominator
NPV = 0.98	(43435-169)*(1-0.98)= 865	(22673-169)*(1-0.98)= 450
NPV = 0.99	(43435-169)*(1-0.99) = 433	(22673-169)*(1-0.99)= 225
NPV = 0.995	(43435-169)*(1-0.995) = 216	(22673-169)*(1-0.995)=113

§However, depends on the NPV (TN/(TN+FN)), and both TN and FN are unknown in this study.

2) **Number of true negatives = (1–prevalence) – number of false positives**

Number of true negatives = (1 – (TP + FP + $xpert_{pos}cult_{missing}$)) – FP) = 1 – TP – 2*FP - $xpert_{pos}cult_{missing}$

Compared to standard specificity formula:

1 = TN + FN + TP + FP

Number of true negatives = 1 – FN – TP – FP

In this case, FN ($xpert_{neg}cult_{pos}$) is not accounted for but instead FP is subtracted a second time in addition to $xpert_{pos}cult_{missing}$, which is essentially the same as done above. As the number of FP (+ $xpert_{pos}cult_{missing}$) is likely to be lower than the number of FN, this might overestimate the number of true negatives.

3) **Number of false positives = number of true positives × (1–PPV)/PPV**

Number of false positives = TP * (1 – (TP / (TP + FP)) / (TP / (TP + FP))

Number of false positives = TP* (FP / (TP + FP)) / (TP / (TP+FP))

Number of false positives = FP

4) **Number of true positives = prevalence x sensitivity**

Number of true positives = ((TP + FP + $xpert_{pos}cult_{missing}$))/denominator) x sensitivity

➔ This calculation multiplies the proportion of Xpert MTB/RIF positive results given the chosen denominator (prevalence) by the probability of returning a positive Xpert MTB/RIF when you have positive sputum culture as found in another study (sensitivity) [4], instead of calculating the proportion of true positives found in the study (proportion TP = TP/(TP+FP))

Method calculation specificity Uganda by Kendall *et al*. (2021) [2]

Specificity = $T_{neg,neg}/(T_{neg,neg} + T_{neg,pos})$

With $T_{neg,neg} = xpert_{neg}cult_{neg}$

With $T_{neg,pos} = xpert_{pos}cult_{neg}$

Specificity = $xpert_{neg}cult_{neg}$ / ($xpert_{neg}cult_{neg}$ + $xpert_{pos}cult_{neg}$)

Specificity = TN / (TN + FP)

Exercise to derive the specificity formula by Kendall *et al*. [2]

1) Kendall *et al*. [2] inferred the sputum culture status of individuals with positive Xpert Ultra but missing culture, estimating the number of FP.

   FP = $(\text{xpert}_{pos}\text{cult}_{neg})_{found}$ + $(\text{xpert}_{trace\_pos}\text{cult}_{missing})$*proportion culture negative among individuals with both results + $(\text{xpert}_{nontrace\_pos}\text{cult}_{missing})$* proportion culture negative among individuals with both results

2) Compared to Ho *et al*. [1], where 22673 out of 43435 consenting participants provided a valid Xpert MTB/RIF result (52%), 12032 out of 12301 consenting participants provided a valid Xpert Ultra result in the study by Kendall *et al*. [2] (98%).

3) Kendall *et al*. [2] estimated the number of true negatives as:

   TN = N + x*U

   → N = number of $\text{xpert}_{neg}\text{cult}_{neg}$

   → U = number of $\text{xpert}_{neg}\text{cult}_{missing}$

   → x = proportion among of $\text{xpert}_{neg}\text{cult}_{missing}$ that would have been cult_{neg} if tested

   Primary; x=99%, in sensitivity analyses: 100%, 99.5%, 98.6%, 94.6%

   Our interpretation

   Using standardized terminology, we would interpret this formula for the estimated number of true negatives as: TN = $(\text{TN})_{confirmed}$ + NPV* $\text{xpert}_{neg}\text{cult}_{missing}$

   The NPV was assumed, since TN is unknown:

   NPV = TN / (TN + FN)

   NPV = cult-xpert- / (cult-xpert- & cult+xpert-)

   (1-prevalence) * N * specificity

   (1-prevalence) * N * specificity + ((prevalence) * N * (1-sensitivity)) [6]

Decision on the final method used in this study

Our method and the method by Kendall *et al*. [2] resulted in the same Xpert Ultra specificity estimate of 99.4% (with trace classified as positive). Since the method by Ho *et al*. [1] resulted in a different specificity estimate, and included more assumptions, we decided not to use the method of Ho *et al*. [1] The reason for not using the method by Kendall *et al*. [2] was because the estimate of culture-positive tuberculosis prevalence ((TP+FN)/individuals consented to screening) when their assumed negative predictive value was used; the resulting culture-positive tuberculosis prevalence estimate applying their primary NPV of 99% might be to high when comparing to culture-positive prevalence (and accompanying specificity) of the prevalence surveys of e.g. Bangladesh (see **Supplementary Table S5**).

Culture-positive tuberculosis prevalence = (TP+FN)/(individuals consented to be screened)*100%

➔ Estimation for TP+FN below (which assumes that individuals consented to be screened but without Xpert screening done are true negative).

1) Culture-positive tuberculosis prevalence Uganda based on calculation Kendall *et al.*:

FN = number of individuals $Xpert_{neg}Cult_{missing}$*(1-NPV) = $Xpert_{neg}Cult_{missing}$*(1-0.99)

- FN if trace as positive: (12032-113)*0.01 = 119.19 FN
  - → % culture-positive tuberculosis: ((35+((12301-113)*0.01))/12301*100) = 1.3%
- FN if trace as positive: (12032-42)*0.01 = 119.9 FN
  - → % culture-positive tuberculosis = ((27+((12032-42)*0.01))/12301*100) = 1.2%

2) Culture-positive tuberculosis prevalence based on this study's method:

Culture-positive TB = TP + FN = (TP/sensitivity(%)*100))

- TP + FN if trace as positive: ((35+(15*0.14)+(4*0.71))/90.9*100)) = 44 TP + FN
  - → % culture-positive tuberculosis = 44/12301*100 = 0.36%
- TP + FN if trace as negative: ((27+(4*0.71))/90.9*100)) = 33 FN
  - → % culture-positive tuberculosis = 33/12301*100 = 0.27%

Table 2. Comparing estimated specificity with the three different methods

<table>
<tr><td rowspan="2">Study data</td><td rowspan="2">Type NAAT results</td><td colspan="3">Specificity calculation</td></tr>
<tr><td>Method Kendall et al. [2]
= TN / (TN + FP)
With TN = N + x*U</td><td>This study's method
= 1 – (FP / (TN+FP))
With TN+FP = (denominator chosen - (TP/sensitivity(%)*100))</td><td>Method Ho et al. [1]
Prevalence = all xpert pos / denominator chosen
Specificity = ((1-prev) - ((prev*sens)*(1-PPV)/PPV)) / (1-prev)</td></tr>
<tr><td rowspan="2">Viet Nam - Ho et al. [1]</td><td>Xpert MTB/RIF pos (denominator = consented)</td><td>TN = 0 + 0.99*(43435-169)=42833
FP = 60 + (0.39*15) = 66

Spec = 42833/(42833+66)
Spec = 0.99846</td><td>Spec = 1 – (66 / (43435 – ((94+(15*0.61))/84.7*100)))
Spec = 0.99848</td><td>Spec = ((1-0.0039) - ((0.0039*0.89)*(1-0.61)/0.61)) / (1-0.0039)
Spec = 0.997772</td></tr>
<tr><td>Xpert MTB/RIF (denominator = valid Xpert result)</td><td>TN = 0 + 0.99*(22673-169) = 22279

Spec = 22279/(22279+66)
Spec =0.9970</td><td>Spec = 1 – (66 / (22673– (94+(15*0.61))/84.7*100)))
Spec = 0.9971</td><td>Spec = ((1-0.0075) - ((0.0075*0.89)*(1-0.61)/0.61)) / (1-0.0075)
Spec = 0.9957</td></tr>
<tr><td rowspan="2">Uganda - Kendall et al. [2]</td><td>Trace as positive (denominator = consented)</td><td>FP = 59+(15*0.86)+(4*0.29)= 73

TN = 0 + 0.99*(12032-113)= 11800

Spec = 11800/(11800+73)
Spec= 0.99385</td><td>Spec = 1 – (73 / (12301– ((35+(15*0.14)+(4*0.71))/82.5*100)))
Spec = 0.9940</td><td>PPV = 35/94 = 0.37
'prevalence' = 113/12301 = 0.0092

Spec = ((1-0.0092) - (0.0092*0.825)*(1-0.37)/0.37) / (1-0.0092)
Spec= 0.986956</td></tr>
<tr><td>Trace as negative (denominator = consented)</td><td>FP = 11 + (4*0.29) = 12
TN = 0 + 0.99*(12032-42)=11870

Spec = 11870/(11870+12)

Spec = 0.998990</td><td>Spec = 1 – (12 / (12301– ((27+(4*0.71))/65.3*100)))
Spec = 0.9990</td><td>PPV = 27/38 = 0.71
'prevalence' = 42/12301 = 0.0034

Spec = ((1-0.0034) - (0.0034*0.653)*(1-0.71)/0.71) / (1-0.0034)
Spec= 0.9991</td></tr>
</table>

References

1. Ho J, Nguyen PTB, Nguyen TA, et al. Reassessment of the positive predictive value and specificity of Xpert MTB/RIF: a diagnostic accuracy study in the context of community-wide screening for tuberculosis. Lancet Infect Dis. **2016**; 16(9):1045–1051.

2. Kendall EA, Kitonsa PJ, Nalutaaya A, et al. The Spectrum of Tuberculosis Disease in an Urban Ugandan Community and Its Health Facilities. Clin Infect Dis Off Publ Infect Dis Soc Am. **2021**; 72(12):e1035–e1043.

3. Marks Guy B., Nguyen Nhung V., Nguyen Phuong T.B., et al. Community-wide Screening for Tuberculosis in a High-Prevalence Setting. N Engl J Med. Massachusetts Medical Society; **2019**; 381(14):1347–1357.

4. Steingart KR, Schiller I, Horne DJ, Pai M, Boehme CC, Dendukuri N. Xpert® MTB/RIF assay for pulmonary tuberculosis and rifampicin resistance in adults - Steingart, KR - 2014 | Cochrane Library. [cited 2025 Jan 12]; . Available from: https://www.cochranelibrary.com/cdsr/doi/10.1002/14651858.CD009593.pub3/full

5. Zifodya JS, Kreniske JS, Schiller I, et al. Xpert Ultra versus Xpert MTB/RIF for pulmonary tuberculosis and rifampicin resistance in adults with presumptive pulmonary tuberculosis. Cochrane Database Syst Rev. **2021**; 2:CD009593.

6. Monaghan TF, Rahman SN, Agudelo CW, et al. Foundational Statistical Principles in Medical Research: Sensitivity, Specificity, Positive Predictive Value, and Negative Predictive Value. Medicina (Mex). **2021**; 57(5):503.
