# Supplementary material for: Mind the Clinic-Community Gap: Re-evaluation of Test Performance and False Positive Results in Community-Wide Tuberculosis Screening
Source: J Infect Dis. 2025 May 23;232(2):e242–6. doi: 10.1093/infdis/jiaf268 (PMC12349932; doi:10.1093/infdis/jiaf268)
Supplement: jiaf268_Supplementary_Data [file jiaf268_supplementary_data.zip › Veeken2025_SupplementaryMaterial.pdf]

Supplementary Material for *Mind the clinic-community gap: re-evaluation of test performance and false positive results in community-wide tuberculosis screening*

Lara D. Veeken, Alvaro Schwalb, Katherine C. Horton, Raspati C. Koesoemadinata, Bachti Alisjahbana, Reinout van Crevel, Rein M.G.J. Houben

Table of contents

S1. METHODS 2

S2. RESULTS 5

Supplementary Table S1. Study characteristics 6

Supplementary Table S2. Community-wide screening Xpert test flow 8

Supplementary Table S3. Community-wide screening positive Xpert and culture availability 8

Supplementary Table S4. Specificity calculation among communities 9

Supplementary Table S5. Study data and calculated specificity and sensitivity 10

Supplementary Table S6. Ratio false positive diagnoses per one true positive diagnosis using study setting's prevalence 11

Supplementary Table S7. Sample size calculation for specificity with a confidence level of (100-α) = 95% and an estimated prevalence of culture-positive tuberculosis of 0.5% 12

Supplementary Table S8. Sample size calculation for sensitivity with a confidence level of (100-α) = 95% and an estimated prevalence of culture-positive tuberculosis of 0.5% 12

References 12

S1. METHODS

First, we comment on our literature review strategy. Second, we outline how we defined terminology to assess the performance of Xpert compared with culture as the reference standard. Third, we define the standard calculations for sensitivity, specificity, and prevalence of culture-positive tuberculosis that are used for the prevalence surveys. Fourth, we define how we performed these calculations for community studies in the absence of true negative (TN) and false negative (FN) numbers. Fifth, we describe how we defined the false positive:true positive (TP:FP) ratio. Sixth, we describe the R packages used for our analyses. Data and analysis code is available at GitHub (https://github.com/ldveeken/ClinicCommunityGap).

Literature review

For the prevalence surveys, we included prevalence surveys included previously by the WHO [1] that tested sputum of all screen-positive individuals with Xpert, and of which data were retraceable. For the community studies, we searched literature available in PubMed, using the search strategy: "(Tuberculosis [MeSH] OR tuberculosis [tiab]) AND (Xpert [tiab]) AND (community [tiab]) AND (Sensitivity and Specificity [MeSH] OR specificity [tiab])". We included studies that tested sputum of an unenriched community population with an Xpert test as initial screening tool for tuberculosis and performed additional sputum culture testing (either of all screened individuals, or only of the individuals with a positive Xpert test), and of which Xpert and culture results of the unenriched community-wide screening population were publicly available. If cohort data was analysed in multiple published articles, only one article was included.

Terminology test performance Xpert

		Reference standard for TB disease	
		Culture positive	Culture negative
Test	Xpert positive	TP	FP
	Xpert negative	FN	TN

Terminology	Abb. 1	Abb. 2	Xpert Ultra result	Culture result (reference)
True negative	TN	$xpert_{neg}cult_{neg}$	Xpert negative	Culture negative
False negative	FN	$xpert_{neg}cult_{pos}$	Xpert negative	Culture positive
True positive	TP	$xpert_{pos}cult_{pos}$	Xpert positive	Culture positive
False positive	FP	$xpert_{pos}cult_{neg}$	Xpert positive	Culture negative

Prevalence surveys: calculations for the specificity, sensitivity and prevalence of culture-positive tuberculosis

Sensitivity	= TP / (TP + FN)
	= $xpert_{pos}cult_{pos}$ / ($xpert_{pos}cult_{pos}$ + $xpert_{neg}cult_{pos}$)
Specificity	= TN / (TN + FP)
	= $xpert_{neg}cult_{neg}$ / ($xpert_{neg}cult_{neg}$ + $xpert_{pos}cult_{neg}$)
Prevalence of culture-positive tuberculosis	= (TP + FN) / all individuals consented
	= ($xpert_{pos}cult_{pos}$ + $xpert_{neg}cult_{pos}$) / all individuals consented

Community studies: calculations for the specificity and prevalence of culture-positive tuberculosis

<u>Specificity</u>
= 1 – probability of returning a positive Xpert test if culture negative
= 1 – (FP / (TN+FP))
With TN+FP = (all individuals consented – (TP/sensitivity(%)*100))

Derivation
Probability returning a positive Xpert test if culture negative = FP / (TN+FP)
= $xpert_{pos}cult_{neg}$ / ($xpert_{neg}cult_{neg}$ + $xpert_{pos}cult_{neg}$)

= number of $xpert_{pos}cult_{neg}{}^{\dagger}$ / estimated number of culture negative individuals
= number of $xpert_{pos}cult_{neg}{}^{\dagger}$ / total number of people consented – estimated number of culture-positives
= number of $xpert_{pos}cult_{neg}{}^{\dagger}$/ (total number of people consented – (TP/sensitivity(%)*100))

†To account for $xpert_{pos}cult_{missing}$ that would have been culture positive/negative if tested, the same method as Kendall *et al.* [2] could be used, by multiplying the number with the proportions found among those tested with both.

<u>Prevalence of culture-positive tuberculosis</u>
= ($TP^{\dagger}$/sensitivity(%))*100
= ($xpert_{pos}cult_{pos}{}^{\dagger}$/sensitivity(%))*100

Calculation of false positive:true positive ratio

Calculating the clinic-based estimates

Ratio = FP/TP, with

FP = (N – (N*(prevalence culture-positive tuberculosis/100)))*(1-specificity)

TP = N*(prevalence culture-positive tuberculosis/100)*sensitivity

Using the point estimates found by Zifodya *et al.* [3]: Xpert Ultra sensitivity 0.909 (95% Credibility Interval [CrI] 0.862 to 0.947) and specificity 0.956 (95% CrI 0.930 to 0.974), and Xpert MTB/RIF sensitivity 0.847 (95% CrI 0.786 to 0.899%) and specificity 0.984 (95% CrI 0.970 to 0.993%)

Calculating ratios found in studies

Ratio = FP/TP

Calculating ratios for studies assuming a culture-positive tuberculosis prevalence of 0.5%

Ratio = FP/TP, with

FP = (N – (N*0.005))*(1-specificity)

TP = N*0.005*sensitivity

For community studies, using calculated specificity and pooled sensitivity found in prevalence surveys (see Table S3)

For prevalence surveys, using calculated sensitivity and specificity (see Table S4)

R packages

The *Hmisc* package was used to calculate sensitivity and specificity with Wilson confidence intervals [4].

The m*ada* package was used to calculate pooled estimates with Wilson confidence intervals [5].

The *epi.ssdxsesp* function of the *EpiR* package was used to perform sample size calculations [6].

S2. RESULTS

Table S1 provides an overview of the study characteristics, displaying similarities and differences between studies. Table S2 and S3 shows Xpert and culture test result availability of the community studies, informing inferring of culture status. Table S4 shows Xpert specificity of community studies with and without inferring culture status, and using clinic or prevalence survey sensitivity in the calculation. We used the more conservative method inferring culture status, and showed that both sensitivity values resulted in the same specificity. Table S5 shows an overview of the test performance of Xpert in all included studies. Table S6 shows the number of false positives diagnoses per one true positive, a) estimated with the clinic-based values based on the found study setting's prevalence of culture-positive tuberculosis, and b) what was reported by the studies. Table S7 and S8 show a sample size calculation for a hypothetical study establishing predefined test performance of Xpert, where the prevalence of sputum culture-positive would be at 500/100k.

Supplementary Table S1. Study characteristics

	WHO prevalence of bacteriologically confirmed tuberculosis (%)	Inclusion criteria eligible participants	Criteria for positive screening	Type and number of culture tests performed	Type and number of Xpert tests performed	Ref
**Xpert MTB/RIF**						
Community						
Viet Nam (2014-15)	0.322	Consenting individuals ≥15 years old	-	Liquid (MGIT)	Xpert MTB/RIF (1x)	[7]
Prevalence surveys						
Bangladesh (2015-16)	0.287	Consenting individuals ≥15 years old	Any lung abnormality on digital CXR or ‘ Symptom-screen positive: Total clinical score ≥3 points<br>Clinical score 1 or 2 with chest X-ray exempted - Cough ≥2 weeks (3 points)<br>Cough <2 weeks (1 point)<br>Haemoptysis in the past month (3 points)<br>Weight loss in the past month (1 point)<br>Fever ≥1 week in the past month (1 point)<br>Night sweats in the past month (1 point))	Solid (Löwenstein-Jensen)<br><br>“Two samples (spot, morning): concentrated preparation, LJ media”	Xpert MTB/RIF (1x)	[1,8]
Kenya (2015-16)	0.558	Consenting individuals ≥15 years old	Cough ≥2 weeks, any lung abnormality on digital CXR, CXR exempted	Solid (Löwenstein-Jensen)<br>“Two samples (spot, morning): concentrated preparation, LJ media”	Xpert MTB/RIF (1x)	[1,8]
Philippines (2016)	1.160	Consenting individuals ≥15 years old	Cough ≥2 weeks and/or haemoptysis, any lung or mediastinum abnormality on digital CXR, CXR exempted	Solid (Ogawa)<br><br>One or two samples, direct preparation, Ogawa media	Xpert MTB/RIF (1x)	[1,8]

Viet Nam (2017-18)	0.322	Consenting individuals ≥15 years old	If the participant had at least one of the following: cough for two weeks or more (or cough of any duration for pregnant women); self-reported TB treatment in the two years preceding the survey; and chest X-ray with abnormalities consistent with TB.	Liquid (BACTEC MGIT 960) and solid (Löwenstein-Jensen)	Xpert MTB/RIF (1x)	[9]
**Xpert Ultra**						
Community						
Uganda (2019)	0.401	Consenting individuals ≥15 years old	-	Liquid (MGIT) and solid (Löwenstein-Jensen)	Xpert Ultra (1x)	[2]
Prevalence surveys						
Treats ZMB (2017-21)	0.638	Consenting individuals ≥15 years old	Those who had a cough for ≥2 weeks or who had ≥2 among 5 'TB suggestive' symptoms (cough of any duration, unexpected weight loss for ≥4 weeks, night sweats for ≥2 weeks, chest pains for ≥2 weeks, fever for ≥2 weeks), or an X-ray score above a predefined threshold (≥40% during the IDP), or who did not have an X-ray done.	Liquid culture (2 tubes)	Xpert Ultra (2x)	[10]
Treats ZAF (2017-21)	0.852					[10]
South Africa (2017-19)	0.852	Consenting individuals ≥15 years old	Participants with any screening symptoms (cough (persistent of any duration), drenching night sweats, unexplained weight loss, and unexplained fever for at least 2 weeks) or a digital chest X-ray classified as abnormal suggestive of tuberculosis, or a combination thereof, and those who did not report symptoms and had no chest X-ray.	Liquid (Bactec MGIT 960)	Xpert Ultra (1x)	[11]
Lesotho (2019)	0.581	Consenting individuals ≥15 years old	Participants who reported any of the four symptoms (i.e., a cough of any duration, fever, unexplained weight loss in the last one month or night sweats) and/or had digital CXR findings suggestive of TB	Liquid (BD BACTEC MGIT) (one tube)	Xpert Ultra (1x)	[12]

Supplementary Table S2. Community-wide screening Xpert test flow

Study	N consented	N provided sputum	N valid Xpert results	N Xpert positive (excluding trace)	Proportion test positive (Xpert positive/valid results [%])
Viet Nam (Ho et al. [7])	43435	23202	22673	169	0.75
Uganda (Kendall et al. [2])					
Salivary	-	-	7947	22	0.28
Non-salivary	-	-	3891	19	0.49
Missing	-	-	194	1	0.52
Total	*12301*	*12231*	*12032*	*42*	*0.35*

Supplementary Table S3. Community-wide screening positive Xpert and culture availability

Study	Positive Xpert and Culture result available			No culture
	TP ($xpert_{pos}cult_{pos}$)	FP ($xpert_{pos}cult_{neg}$)	Total	**($xpert_{pos}cult_{neg}$)**
Viet Nam (Ho et al. [7])	94 (61%)	60 (39%)	154 (100%)	15
Uganda (Kendall et al. [2])				
Trace	8 (14%	48 (86%)	56 (100%)	15
Higher than trace	27 (71%)	11 (29%)	38 (100%)	4
All Xpert positive	35 (37%)	59 (63%)	94 (100%)	19

Supplementary Table S4. Specificity calculation among communities

	Not inferring missing cultures		Inferring missing cultures	
**Study**	Calculation	Specificity (95% CI)	Calculation	Specificity (95% CI)
**Viet Nam (Xpert MTB/RIF)**	FP = 60		FP = 60 + (0.39*15) = 66	
Sensitivity = 84.7% (clinic **[3]**)	1 – (60 / (43435 – ((94/84.7*100)))<br>1 – (60 / (43435 –111))<br>1 – (60 /43324)	0.999 (0.998-0.999)	1 – (66 / (43435 – ((94+(15*0.61))/84.7*100)))<br>1 – (66 / (43435 – 122))<br>1 – (66 / 43313)	0.998 (0.998-0.999)
Sensitivity = 73.2% (pooled prevalence surveys)	1 – (60 / (43435 – ((94/73.2*100)))<br>1 – (60 / (43435 – 128))<br>1 – (60 / 43307)	0.999 (0.998-0.999)	1 – (66 / (43435 – ((94+(15*0.61))/73.2*100)))<br>1 – (66 / (43435 – 141))<br>1 – (66 / 43294)	0.998 (0.998-0.999)
**Uganda (Xpert Ultra)**				
**Trace as positive**	FP = 59		FP = 59+(15*0.86)+(4*0.29)= 73	
Sensitivity = 90.9% (clinic [3])	1 – (59 / (12301– (35/90.9*100))<br>1 – (59 / (12301 - 39))<br>1 – (59 / 12262)	0.995 (0.994-0.996)	1 – (73 / (12301– ((35+(15*0.14)+(4*0.71))/90.9*100)))<br>1 – (73 / (12301 - 44))<br>1 – (73 / 12257)	0.994 (0.993-0.995)
Sensitivity = 82.5% (pooled prevalence surveys)	1 – (59 / (12301– (35/82.5*100))<br>1 – (59 / (12301 - 42))<br>1 – (59 / 12259)	0.995 (0.994-0.996)	1 – (73 / (12301– ((35+(15*0.14)+(4*0.71))/82.5*100)))<br>1 – (73 / (12301 - 48))<br>1 – (73 / 12253)	0.994 (0.993-0.995)
**Trace as negative**	FP = 11		FP = 11 + (4*0.29) = 12	
Sensitivity = 90.9% (clinic [3])	1 – (11 / (12301– (27/90.9*100)))<br>1 – (11 / (12301 – 30))<br>1 – (11 / 12271)	0.999 (0.999-1.000)	1 – (12 / (12301– ((27+(4*0.71))/90.9*100)))<br>1 – (12 / (12301 – 33))<br>1 – (12 / 12268)	0.999 (0.998-1.000)
Sensitivity = 65.3% (pooled prevalence surveys)	1 – (11 / (12301– ((27/65.3*100)))<br>1 – (11 / (12301–41))<br>1 – (11 / 12260)	0.999 (0.999-1.000)	1 – (12 / (12301– ((27+(4*0.71))/65.3*100)))<br>1 – (12 / (12301–46))<br>1 – (12 / 12255)	0.999 (0.998-1.000)

Calculation 95% CI specificity:

$SE = \sqrt{\frac{specificity\ x\ (1-specificity)}{N}}$ with N=TN+FP

95% CI = specificity -/+ 1.96*SE.

Rationale calculating specificity among individuals consented:

We assume that sputum quality control was stricter in the Viet Nam study than in Uganda, and most of salivary samples were excluded in Viet Nam but not in Uganda (see Table S2). Based on available data, we assume that the estimate among "individuals consented' is closer to the true specificity of analysing all samples, and thereby comparable to the Uganda (excluding trace) data, than an estimate among "individuals with a valid Xpert result". As the specificity estimated among "individuals consented" is assumed to reduce to 0.997 only if the Xpert test positivity rate among non-analysed samples in Viet Nam would have been ≥70% of that among the analysed samples, which we assumed unlikely**.**

Supplementary Table S5. Study data and calculated specificity and sensitivity

	Eligible study population	Participants	Individuals screened positive	TP	FP	TN	FN	Specificity (95% CI)[a]	Sensitivity (95% CI)[b]	Ref
**Xpert MTB/RIF**										
Community										
Viet Nam (2014-15)	51,136	43,435	-	94	60	-	-	0.998 (0.998-0.999)	-	[7]
Prevalence surveys										
Bangladesh (2015-16)	108,834	98,710	20,594	132	137	20,134	22	0.993 (0.992-0.994)	0.857 (0.793-0.904)	[1,8]
Kenya (2015-16)	76,291	63,050	9,715	152	81	8,045	69	0.990 (0.988-0.992)	0.688 (0.624-0.745)	[1,8]
Philippines (2016)	61,466	46,689	18,597	159	228	15,275	73	0.985 (0.983-0.987)	0.685 (0.623-0.742)	[1,8]
Viet Nam (2017-18)	87,207	61,763	4,738	130	74	3,926	60	0.982 (0.977-0.985)	0.684 (0.615-0.746)	[9]
**Xpert Ultra**										
Community										
Uganda (2019)										[2]
Trace as positive	34,135	12,301	-	35	59	-	-	0.994 (0.993-0.995)	-	
Trace as negative	34,135	12,301	-	27	11	-	-	0.999 (0.998-1.000)	-	
Prevalence surveys										
Treats ZMB (2017-21)										[10]
Trace as positive	28,262	8,922	1,587	36	14	626	5	0.978 (0.964-0.987)	0.878 (0.745-0.947)	
Trace as negative	28,262	8,922	1,587	34	8	632	7	0.988 (0.976-0.994)	0.829 (0.687-0.915)	
Treats ZAF (2017-21)										[10]
Trace as positive	45,598	2,048	491	19	15	291	3	0.951 (0.921-0.970)	0.864 (0.667-0.953)	
Trace as negative	45,598	2,048	491	12	9	297	10	0.971 (0.945-0.984)	0.545 (0.347-0.731)	
South Africa (2017-19)										[11]
Trace as positive	53,250	3,5191	9,066	170	104	6,567	48	0.984 (0.981-0.987)	0.780 (0.720-0.830)	
Trace as negative	53,250	3,5191	9,066	144	66	6,605	74	0.990 (0.987-0.992)	0.661 (0.595-0.720)	
Lesotho (2019)										[12]
Trace as positive	26,857	21,719	7,584	86	121	6,369	22	0.981 (0.978-0.984)	0.796 (0.711-0.861)	
Trace as negative	26,857	21,719	7,584	72	66	6,424	36	0.990 (0.987-0.992)	0.667 (0.573-0.748)	

[a] Specificity: for community = see page 3; for prevalence surveys: TN/(TN+FP). 95% CI: specificity -/+ 1.96*SE. With Wilson confidence intervals **[4]**

[b] Sensitivity = TP/(TP+FN). 95% CI: specificity -/+ 1.96*SE. With Wilson confidence intervals **[4]**

[†] 4197 with Xpert and culture available. TN = 4197-7 culture missing/NTM/contaminated – 130 TP – 74 FP – 60 FN = 3926

Abbreviations: CI – confidence interval; FN – False Negative; FP – False Positive; ref – reference; TN – True Negative; TP – True Positive; ZAF – South Africa; ZMB – Zambia

Supplementary Table S6. Ratio false positive diagnoses per one true positive diagnosis using study setting's prevalence

		Estimated ratio using clinic-estimated[b]		Ratio found in studies[c]	
	**Prevalence of culture-positive tuberculosis (%)[a]**	**Ratio FPs per 1 TP**	**Proportion false positives of all Xpert positive (%)**	**Ratio FPs per 1 TP**	**Proportion false positives of all Xpert positive (%)**
**Xpert MTB/RIF**					
Community					
Viet Nam (2014-15)	0.3	6.28	86	0.64	39
Prevalence surveys					
Bangladesh (2015-16)	0.7	2.51	71	1.04	51
Kenya (2015-16)	2.3	0.81	45	0.53	35
Philippines (2016)	1.2	1.50	60	1.43	59
Viet Nam (2017-18)	4.0	0.45	31	0.57	36
**Xpert Ultra**					
Community					
Uganda (2019)					
Trace as positive	0.4	12.05	92	1.83	65
Trace as negative	0.3	-	-	0.41	29
Prevalence surveys					
Treats ZMB (2017-21)					
Trace as positive	2.6	1.83	65	0.39	28
Trace as negative	2.6	-	-	0.24	19
Treats ZAF (2017-21)					
Trace as positive	4.5	1.03	51	0.79	44
Trace as negative	4.5	-	-	0.75	43
South Africa (2017-19)					
Trace as positive	2.4	1.96	66	0.61	38
Trace as negative	2.4	-	-	0.46	31
Lesotho (2019)					
Trace as positive	1.4	3.35	77	1.41	58
Trace as negative	1.4	-	-	0.92	48

[a] For community: (TP+FN)/(individuals consented to be screened)*100% with TP+FN = (TP/sensitivity(%)*100)); for prevalence surveys: (TP+FN)/(individuals screened positive)*100

[b] FP = (N – (N*(prevalence culture-positive tuberculosis/100)))*(1-specificity)); TP = N*(prevalence culture-positive tuberculosis/100)*sensitivity

Using the point estimates found by Zifodya *et al.* [3]: Xpert Ultra sensitivity 90.9% and specificity 95.6% & Xpert MTB/RIF sensitivity 84.7% and specificity 98.4%.

[c] To account for $xpert_{pos}cult_{missing}$ in community studies, that would have been culture positive/negative if tested, the same method as Kendall *et al.* [2] could be used, by multiplying the number with the proportions found among those tested with both.

Abbreviations: CI – confidence interval; FN – False Negative; FP – False Positive; ref – reference; TN – True Negative; TP – True Positive; ZAF – South Africa; ZMB – Zambia

Supplementary Table S7. Sample size calculation for specificity with a confidence level of (100-α) = 95% and an estimated prevalence of culture-positive tuberculosis of 0.5%

	Expected specificity					
**95% CI width**	**99.4%**	**99.5%**	**99.6%**	**99.7%**	**99.8%**	**99.9%**
± 0.1	23,026	19,208	15,382	11,548	7,707	3,857
± 0.2	5,757	4,802	3,846	2,887	1,927	NA
± 0.3	2,559	2,135	1,710	1,284	NA	NA

Supplementary Table S8. Sample size calculation for sensitivity with a confidence level of (100-α) = 95% and an estimated prevalence of culture-positive tuberculosis of 0.5%

	Expected sensitivity						
**95% CI width**	**60%**	**65%**	**70%**	**75%**	**80%**	**85%**	**90%**
± 0.1	184,390,024	174,786,377	161,341,271	144,054, 706	122,926,683	97,957,200	69,146,259
± 0.5	7,375,601	6,991,456	6,453,651	5,762,189	4,917,068	3,918,288	2,765,851
± 1	1,843,901	1,747,864	1,613,413	1,440,548	12,29,267	979,572	691,463
± 2	460,976	436,966	403,354	360,137	307,317	244,893	172,866
± 5	73,757	69,915	64,537	57,622	49,171	39,183	27,659

References

1. National tuberculosis prevalence surveys: what diagnostic algorithms should be used in future? [Internet]. [cited 2024 Oct 11]. Available from: https://www.who.int/publications/i/item/9789240073913

2. Kendall EA, Kitonsa PJ, Nalutaaya A, et al. The Spectrum of Tuberculosis Disease in an Urban Ugandan Community and Its Health Facilities. Clin Infect Dis Off Publ Infect Dis Soc Am. **2021**; 72(12):e1035–e1043.

3. Zifodya JS, Kreniske JS, Schiller I, et al. Xpert Ultra versus Xpert MTB/RIF for pulmonary tuberculosis and rifampicin resistance in adults with presumptive pulmonary tuberculosis. Cochrane Database Syst Rev. **2021**; 2:CD009593.

4. Jr FEH, functions) CD (contributed several functions and maintains latex. Hmisc: Harrell Miscellaneous [Internet]. 2024 [cited 2025 Jan 8]. Available from: https://cran.r-project.org/web/packages/Hmisc/index.html

5. Sousa-Pinto PD with contributions from B. mada: Meta-Analysis of Diagnostic Accuracy [Internet]. 2022 [cited 2024 Nov 22]. Available from: https://cran.r-project.org/web/packages/mada/index.html

6. epiR (version 2.0.76) [Internet]. [cited 2024 Dec 6]. Available from: https://www.rdocumentation.org/packages/epiR/versions/2.0.76/topics/epi.about

7. Ho J, Nguyen PTB, Nguyen TA, et al. Reassessment of the positive predictive value and specificity of Xpert MTB/RIF: a diagnostic accuracy study in the context of community-wide screening for tuberculosis. Lancet Infect Dis. **2016**; 16(9):1045–1051.

8. National tuberculosis prevalence surveys 2007-2016 [Internet]. [cited 2024 Oct 11]. Available from: https://www.who.int/publications/i/item/9789240022430

9. Nguyen HV, Tiemersma EW, Nguyen HB, et al. The second national tuberculosis prevalence survey in Vietnam. PloS One. **2020**; 15(4):e0232142.

10. Floyd S, Klinkenberg E, Haas P de, et al. Optimising Xpert-Ultra and culture testing to reliably measure tuberculosis prevalence in the community: findings from surveys in Zambia and South Africa. BMJ Open. **2022**; 12(6):e058195.

11. Moyo S, F I, M V der W, et al. Prevalence of bacteriologically confirmed pulmonary tuberculosis in South Africa, 2017-19: a multistage, cluster-based, cross-sectional survey. Lancet Infect Dis [Internet]. **2022** [cited 2024 Oct 11]; 22(8). Available from: https://pubmed.ncbi.nlm.nih.gov/35594897/

12. Matji R, Maama L, Roscigno G, et al. Policy and programmatic directions for the Lesotho tuberculosis programme: Findings of the national tuberculosis prevalence survey, 2019. PloS One. **2023**; 18(3):e0273245.
